# Causal relationship between obesity and meniscal injuries: Two-sample Mendelian randomization

**DOI:** 10.1097/MD.0000000000036510

**Published:** 2023-12-01

**Authors:** Gaung-hua Deng

**Affiliations:** a Ya’an Hospital of Traditional Chinese Medicine

**Keywords:** Mendelian randomization, meniscal injury, obesity

## Abstract

To investigate the causal relationship between obesity and meniscal injuries using Mendelian randomization (MR). Genetic loci independently associated with obesity and meniscal injuries in people of European origin were selected as instrumental variables using pooled data from genome-wide association studies. Three MR analyses, MR-Egger, weighted median and inverse variance weighting, were used to investigate the causal relationship between obesity and meniscal injuries. The results were tested for robustness by heterogeneity and multiplicity tests, and sensitivity analyses were performed using the “leave-one-out” method. The inverse variance weighting results showed an OR (95% CI) of 1.13 (1.04–1.22), *P* = .003, indicating a causal relationship between obesity and the occurrence of meniscal injuries. And no heterogeneity and multiplicity were found by the test and sensitivity analysis also showed robust results. In this study, genetic data were analyzed and explored using 2-sample MR analysis, and the results showed that obesity is a risk factor for meniscal injuries.

## 1. Introduction

The meniscus is an important component within the knee joint, the meniscus stabilizes the knee joint, transmits knee loading forces and promotes intra-articular nutrition.^[1]^ It is the load stabilizing role played by the meniscus that ensures that the knee joint is protected from injury over years of weight-bearing exercise.^[2]^ When the meniscus is damaged, the structure is disturbed and the knee joint will not be able to move normally. It has been found that the 2 most common causes of meniscal injuries are acute trauma and degenerative diseases.^[3,4]^ Due to the improved living conditions and increased energy intake nowadays, there are more and more obese people.^[5,6]^ However, metabolic disorders in obese individuals can cause various complications.^[7,8]^ Some studies have suggested that obese subjects may be more prone to meniscal injuries than normal weight subjects, but conclusions have been mixed.^[9,10]^ Therefore, the causal relationship between obesity and meniscal injuries still needs further investigation.

The association between obesity and meniscal injuries may be influenced to some extent by confounders and reverse causality inherent in traditional observational studies.^[11]^ In contrast, Mendelian randomization (MR), a genetic epidemiological method, is a useful tool for assessing the causal role of obesity and meniscal injuries.^[12]^ By using genetic variants such as single nucleotide polymorphism (SNP) as instrumental variants that can modify disease risk factors or exposures, MR studies can enhance causal inference of exposure-outcome associations.^[13]^ According to Mendel laws of inheritance, genetic variants are not susceptible to confounding factors because they are randomly assigned during gamete formation.^[14]^ In addition, confounders and reverse causality can be minimized as genotypes cannot change as the disease progresses.^[15]^

To this end, we conducted a 2-sample MR study to examine the causal relationship between obesity and meniscal injuries. We aimed to provide significant evidence for the causal role of obesity in causing meniscal injuries.

## 2. Data and methods

### 2.1. Data source

Genome-wide association study (GWAS) data on obesity and meniscal injury were obtained via the IEU OpenGWAS project (mr cieu.ac.uk) website. The website was accessed on 2023-08-19. The population source for all final data was European, male and female. Including obesity (ieu-a-90) containing 2380,428 SNPs with a sample size of 98,697 and meniscal injuries (finn-b-M13_MENISCUSDERANGEMENTS) containing 16,380,200 SNPs, with 13,568 individuals in the trial group and 147,221 in the control group. This study was a re-analysis of previously collected and published publicly available data and therefore did not require additional ethical approval.

### 2.2. Conditioning of SNP as an instrumental variable

First, the instrumental variables were highly correlated with exposure, with F > 10 as a strong correlation criterion.^[16]^ Second, the instrumental variable is not directly related to the outcome, but only affects the outcome through exposure, i.e., there is no genetic pleiotropy. In this study, the nonexistence of genetic pleiotropy was indicated by a non-zero intercept term (*P* < .05) in the MR-Egger regression model.^[17]^ Finally, instrumental variables were not associated with untested confounding.^[18]^ The human genotype-phenotype association database Phenoscanner V2 was searched for phenotypes associated with the instrumental variables at the genome-wide significance level to determine whether these SNPs were associated with potential risk factors.^[19]^

### 2.3. SNP screening

Significant SNPs were screened from the pooled GWAS data for obesity (with *P* < 5 × 10^−8^ as the screening condition)^[20]^; the chain imbalance coefficient r^2^ was set to be 0.001, and the width of the chain imbalance region to be 10,000 kb to ensure that the individual SNPs were independent of each other.^[21]^ The obesity-related SNPs screened above were extracted from the GWAS pooled data of meniscal injuries, while SNPs directly related to outcome indicators were excluded (*P* < 5 × 10^−8^). The F value of each SNP was calculated, and SNPs with weak instrumental variables (F value <10) were excluded.^[22]^ And the human genotype-phenotype association database was queried to screen for potentially relevant risk factor SNPs and exclude them.^[23]^

### 2.4. Causality validation methods

The causal relationship between exposure (obesity) and outcome (meniscus injury) was mainly verified using inverse variance weighted (IVW) as, supplemented by 3 MR analysis methods, namely MR-Egger and weighted median (WME), with SNPs as instrumental variables.

### 2.5. Sensitivity analysis

Sensitivity analyses were performed using several methods. First, the Cochran *Q* test was used to assess the heterogeneity among the individual SNP estimates, and a statistically significant Cochran *Q* test proved that the analyses were significantly heterogeneous. Second, MR pleiotropy residual sum and outlier (MR-PRESSO) was used to validate the results in the IVW model, to correct for the effect of outliers, and if outliers existed, they were removed and the analysis was repeated. Third, the horizontal multiplicity of SNPs was tested using the MR Egger intercept test (MR Egger intercept test), and if the intercept term in the MR Egger intercept test analysis was statistically significant, it indicated that the MR analysis had significant horizontal multiplicity. Fourth, “leave-one-out” sensitivity analyses were performed by removing a single SNP at a time to assess whether the variant drove the association between the exposure and outcome variables. Fifth, funnel plots and forest plots were constructed to visualize the results of the sensitivity analyses. *P* < .05 suggests that there is a potential causal relationship in the MR analyses, which is statistically significant. All statistical analyses were performed using the “TwoSampleMR” package in R software version 4.3.0.

## 3. Results

### 3.1. Instrumental variables

Seventeen SNPs that were strongly associated with obesity (*P* < 5 × 10^−8^) without chain imbalance (r^2^ < 0.001, kb = 10,000) were screened in the current study. 17 SNPs remained by taking the intersection with SNPs in the pooled GWAS data for meniscal injuries and also by excluding SNPs that were directly associated with the outcome metrics in our study, the F value of each SNP was >10, indicating the absence of weak instrumental variables (Table 1). We searched the human genotype-phenotype association database and found no potentially relevant risk factor SNPs.

**Table 1:** Information on the final screening of obesity SNPs from GWAS data (n = 17).

ID	SNP	Effect_Allele	Other_Allele	β	SE	*P*	F
1	rs10182181	G	A	0.073	0.012	3.30E-09	37
2	rs11075989	T	C	0.21	0.012	5.00E-67	306
3	rs13130484	T	C	0.11	0.013	3.40E-16	71
4	rs13393304	G	A	0.18	0.017	9.50E-27	112
5	rs2030323	C	A	0.11	0.015	2.50E-12	53
6	rs2307111	C	T	-0.069	0.013	3.30E-08	28
7	rs29939	G	A	0.072	0.013	4.20E-08	30
8	rs4929923	C	T	0.075	0.013	8.00E-09	33
9	rs523288	T	A	0.13	0.015	1.80E-19	75
10	rs527248	G	A	0.11	0.016	6.30E-13	47
11	rs7138803	A	G	0.084	0.013	2.60E-11	41
12	rs7141420	T	C	0.079	0.012	2.30E-10	43
13	rs7531118	C	T	0.08	0.013	4.60E-10	37
14	rs8028313	G	C	-0.1	0.015	1.70E-11	44
15	rs887912	C	T	-0.082	0.014	2.30E-09	34
16	rs9816226	T	A	0.11	0.016	1.00E-11	47
17	rs987237	G	A	0.13	0.016	1.00E-16	66

GWAS = genome-wide association study, SNP = single nucleotide polymorphism.

### 3.2. Causal relationship between obesity and meniscal injuries

**Figure 1.**
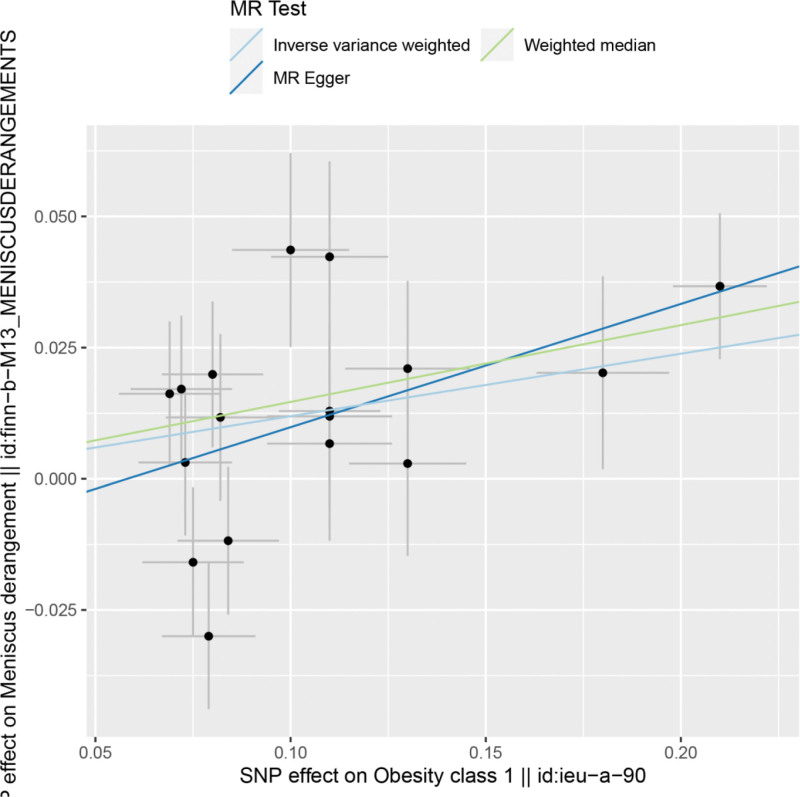
Scatter plot of obesity and meniscal injuries.

**Figure 2.**
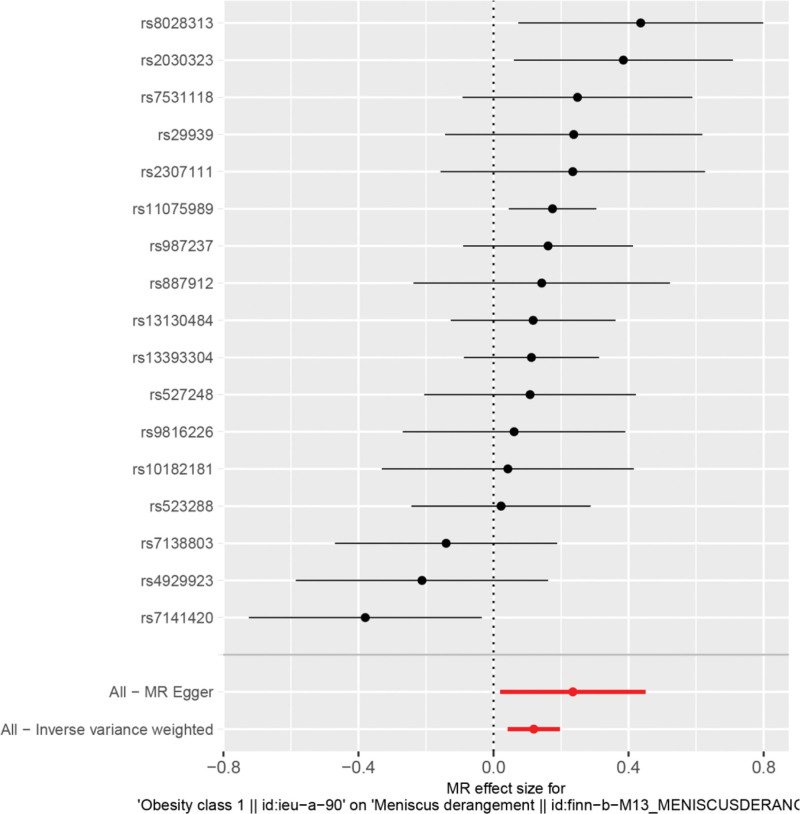
Forest plot of obesity and meniscal injuries.

By MR analysis, IVW, WME and MR-Egger results all showed that obesity was causally associated with meniscal injuries. IVW:OR = 1.13, 95% CI = 1.04 to 1.22, *P* = .003; WME:OR = 1.18, 95% CI = 1.05 to 1.27, *P* = .002; MR-Egger: OR = 1.26, 95% CI = 1.02 to 1.57, *P* = .049 (Table 2). We can see from both the scatter plot (Fig. 1) and the forest plot (Fig. 2) that there is a causal relationship between obesity and meniscal injuries.

**Table 2:** MR regression results of the 3 methods.

Method	β	SE	OR (95% CI)	*P*
IVW	0.119	0.039	1.13 (1.04–1.22)	.003
WME	0.146	0.048	1.18 (1.05–1.27)	.002
MR-Egger	0.235	0.110	1.26 (1.02–1.57)	.049

IVW = inverse variance weighting, MR = Mendelian randomization, WME = weighted median.

### 3.3. Sensitivity analysis

**Figure 3.**
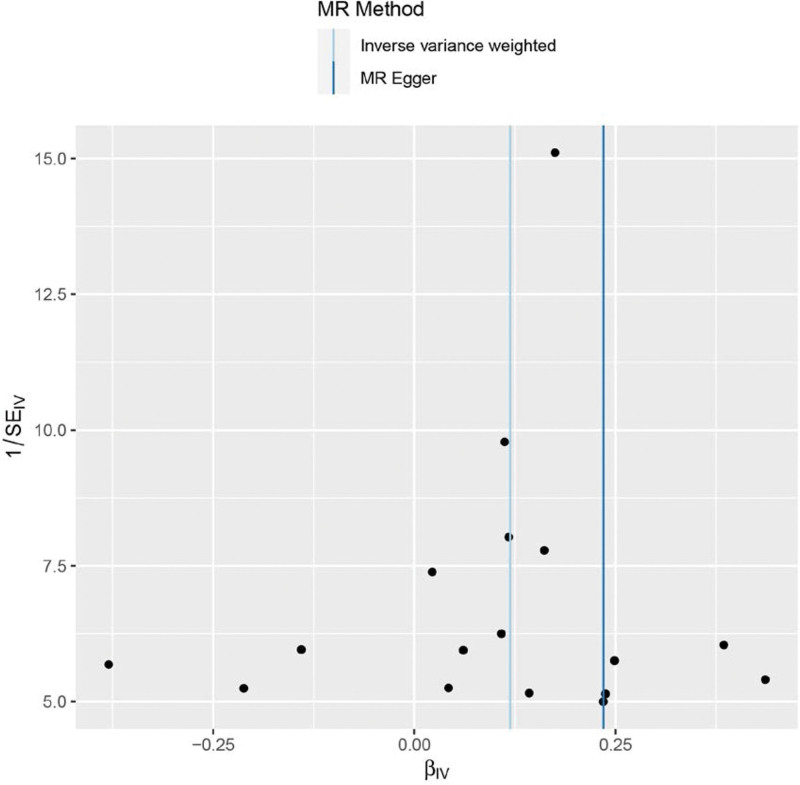
Funnel plot of obesity and meniscal injuries.

**Figure 4.**
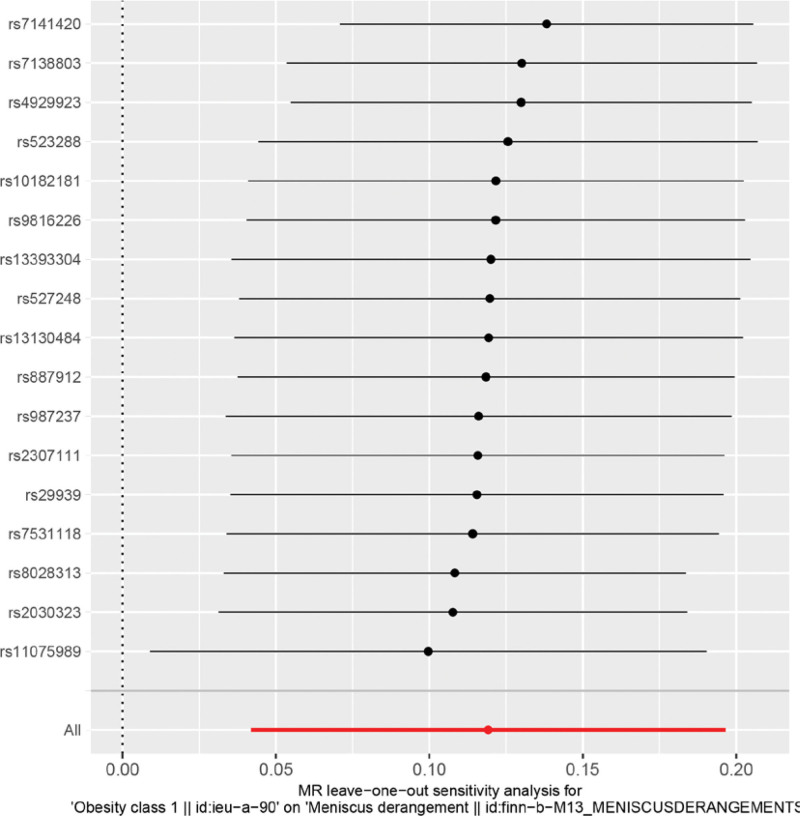
Analysis of obesity and meniscal injuries by the leave-one-out method.

Heterogeneity was tested using the IVW method (Cochran *Q* test, *P* = .148), and the results suggested that there was no heterogeneity. A funnel plot was drawn to show the heterogeneity results, as shown in Figure 3. The use of MR-PRESSO was used to screen SNPs that could lead to heterogeneity, and the results did not reveal any SNPs that would lead to heterogeneity in the results. The Global test results by MR-PRESSO suggested no pleiotropy (*P* = .277). The “leave-one-out” method uses the IVW method by default, and as can be seen in Figure 4, no single SNP will have a large impact on the overall results after eliminating any SNP, indicating that the results are robust.

## 4. Discussion

It is known that obesity may be an observational risk factor for meniscal injuries, but the causality of this association is unclear. Our MR study aimed to reveal the causal relationship between obesity and meniscal injury. The results showed a causal association between obesity and the occurrence of meniscal injuries by 2-sample MR results, with an OR (95 % CI) of 1.13 (1.04–1.22), *P* = .003, suggesting that the probability of meniscal injuries is greater in people with obese stature compared to the general population.

Rohde^[9]^ found that meniscal injuries usually occur in a higher population of obese patients by retrospectively analyzing 1185 cases of meniscal injuries in a population of children and adolescents.

Rai^[24]^ found that transcripts related to oxygen transport, calcium binding and cellular homeostasis were elevated with elevated obesity, while transcripts related to extracellular matrix deposition, cell migration and glucosamine metabolic processes were suppressed. Failure of extracellular matrix deposition and increased calcium binding may contribute to meniscal injury.

However, Giordano^[10]^ found that high obesity was not statistically associated with the development of meniscal injuries by retrospectively analyzing 489 patients with meniscal injuries between 2011 and 2021.

The present study confirms a causal relationship between obesity and the occurrence of meniscal injuries from a genetic perspective. The results of the present study are consistent with the findings of Rohde and Rai that obesity is a risk factor for the development of meniscal injuries and that high obesity increases the incidence of meniscal injuries. Giordano retrospective study found that obesity did not affect the incidence of meniscal injuries. This may be due to the fact that retrospective studies are susceptible to confounding factors and reverse causality, and therefore the causal inferences drawn are considered to be of limited value. MR analysis is a new epidemiological approach that utilizes genetic variation as an instrumental variable for exposure to strengthen causal inferences. This approach reduces the effects caused by confounding factors.^[25]^

At the same time this study has some limitations. Firstly, as all the data are from people of European origin, the results are not representative of a truly random population sample, nor are they applicable to other so races. Secondly, although various sensitivity analyses have been performed in this study to test the hypotheses of the MR study, it is difficult to completely rule out horizontal pleiotropy of instrumental variables. Finally, the current sample size of GWAS data is still not large enough, and more in-depth studies using more GWAS data are needed in the future.

## 5. Conclusion

In conclusion, this study analyzed and explored the genetic data using 2-sample MR analysis, and the results showed that there is a causal relationship between obesity and the occurrence of meniscal injuries.

## Author contributions

**Conceptualization:** Gaung-hua Deng.

**Data curation:** Gaung-hua Deng.

**Formal analysis:** Gaung-hua Deng.

**Funding acquisition:** Gaung-hua Deng.

**Investigation:** Gaung-hua Deng.

**Methodology:** Gaung-hua Deng.

**Project administration:** Gaung-hua Deng.

**Resources:** Gaung-hua Deng.

**Software:** Gaung-hua Deng.

**Supervision:** Gaung-hua Deng.

**Validation:** Gaung-hua Deng.

**Visualization:** Gaung-hua Deng.

**Writing – original draft:** Gaung-hua Deng.

**Writing – review & editing:** Gaung-hua Deng.
